# The Role of Global Longitudinal Strain in the Follow-Up of Asymptomatic Patients with Chronic Primary Mitral Regurgitation

**DOI:** 10.3390/jcm13175304

**Published:** 2024-09-07

**Authors:** Catalina Ileana Badau Riebel, Rares Ilie Orzan, Andra Negru, Lucia Agoston-Coldea

**Affiliations:** 1 Department of Cardiology, “Iuliu Hatieganu” University of Medicine and Pharmacy, 400347 Cluj Napoca, Romania; agoston.lucia@umfcluj.ro; 2 Department of Internal Medicine, “Iuliu Hatieganu” University of Medicine and Pharmacy, 400347 Cluj Napoca, Romania; orzan.rares@umfcluj.ro (R.I.O.); negru.andra@umfcluj.ro (A.N.); 3 2nd Department of Internal Medicine, Emergency County Hospital, 400347 Cluj-Napoca, Romania

**Keywords:** primary mitral regurgitation, global longitudinal strain, multimodality imaging

## Abstract

**Background/Objectives**: In patients with chronic primary mitral regurgitation (MR), postoperative persistent left ventricular (LV) dysfunction underlines the lack of a sensitive parameter that can identify subclinical LV dysfunction and optimize the timing of intervention. Left ventricular global longitudinal strain (LV-GLS) is a measure of the longitudinal left ventricular systolic function, with prognostic significance. Its role in the follow-up of asymptomatic patients with MR is, however, poorly defined. The aim of this study was to assess the relative changes in LV-GLS in a cohort of MR patients and to correlate these changes with the need for intervention during a follow-up period. **Methods**: We conducted a prospective study on a cohort of 218 patients, divided into three subgroups according to MR severity (mild, moderate, severe). LV-GLS was measured at baseline and every six months during a median follow-up period of 30 months. The composite endpoint was the occurrence of heart failure symptoms, hospitalization for heart failure, LVEF < 60%, LVEDD > 45 mm, new onset atrial fibrillation, or cardiovascular death. **Results**: Patients with moderate and severe MR had a significantly lower GLS at baseline than those with mild MR (19.5% and 19.1% versus 22.3%, *p* < 0.01) despite a normal LVEF in all subgroups. The relative decrease in LV-GLS occurred earlier (at 12 months vs. 24 months) and was more evident in patients with moderate and severe MR (13.6% and 14.5%, respectively) versus patients with mild MR (6.72%). The baseline LV-GLS being under 18% and a relative decrease of over 10% in GLS were independent predictors of a composite outcome (HR = 1.59, CI 95% 1.17–2.86; HR = 1.74, CI 95% 1.2–2.91, *p* < 0.01). **Conclusions**: LV-GLS is a valuable monitoring tool for asymptomatic MR patients, a relative decrease > 10% in GLS may be predictive for the need for valve intervention.

## 1. Introduction

In order to correct the hemodynamic impact of a severely regurgitant mitral valve (MV) on the left ventricle (LV) and improve long term prognosis, the mainstay of management involves surgical replacement or repair of the MV.

Current guidelines recommend mitral valve surgery for symptomatic patients with severe MR or asymptomatic patients with echocardiographic proof of LV dysfunction, currently defined as a left ventricular ejection fraction (LVEF) under 60%, or LV dilation dilatation (left ventricular end systolic diameter, LVESD > 45 mm), or in cases of pulmonary arterial hypertension or newly onset atrial fibrillation [1].

These criteria, however, raise several challenges in the management of patients with severe primary MR.

Firstly, the LVEF, in the presence of severe MR, does not accurately reflect LV systolic function, as its calculation does not take into account the fact that the LV stroke volume consists of the aortic forward flow and the regurgitant flow into the left atrium. Consequently, patients with irreversible myocardial damage secondary to chronic volume overload may be falsely perceived as having a “normal” ejection fraction [1].

Secondly, it the absence of the recommended criteria for intervention guidance for choosing between watchful waiting and early intervention is lacking [2,3]. None of the parameters used for intervention can predict disease progression, although there is evidence that better outcomes in terms of survival and preservation of LV function are achieved with early intervention [4,5,6].

Therefore, alternative parameters are needed to detect reversible myocardial damage in its early stages and inform on the appropriate timing of intervention in order to avoid irreversible myocardial dysfunction and improve long-term prognosis. Left ventricular global longitudinal strain (LV-GLS), measured using speckle tracking echocardiography, is currently considered an early, sensitive marker of LV dysfunction, with superior prognostic value to that of the LVEF for predicting major cardiac events [7]. This was explained by an initial loss of function in the longitudinal fibers, with a compensatory increase in the circumferential shortening, resulting in a preserved LVEF [8].

The role of LV-GLS was assessed in valvular and nonvalvular heart disease.

In patients with non-ischemic dilated cardiomyopathy and significant secondary MR, patients with severe MR had a more impaired LV-GLS than those with mild MR, despite similar values of the LVEF, suggesting that LV-GLS may be a more sensitive parameter for LV systolic dysfunction [9].

In patients with secondary MR, impaired LV-GLS was associated with an increased risk of all-cause mortality, whereas the LVEF was not [10].

The overestimation of the LV systolic function by the LVEF was emphasized by two large trials investigating the benefit of MV intervention in patients with secondary MR [11,12]. Transcatheter MV treatment did not add significant benefits to optimal medical therapy for patients in the MITRA-FR trial, whereas in the COAPT trial, MV intervention resulted in a significantly lower rate of heart failure hospitalization and all-cause mortality. It was suggested that this difference in outcome was caused by a more advanced LV disease in the MITRA-FR trial despite a similar baseline LVEF in both study populations. Zho et al. showed that LV-GLS was independently associated with survival outcomes in asymptomatic patients with moderate aortic stenosis and preserved LVEFs, a reduced LV-GLS (<15.2%) being associated with poor survival outcomes, even after aortic valve replacement [13]. Similar data were reported by Stassen et al., who found that a LV-GLS less than 16% was associated with an increase in all-cause mortality and the need for aortic valve replacement [14].

Similarly, in patients with aortic regurgitation, a worse LV-GLS was associated with disease progression and the need for aortic valve replacement, with cutoff points varying between −17.4 and −19.3% [15,16,17,18,19].

Several studies have shown that preoperative GLS in primary MR patients undergoing surgery is the most accurate parameter in detecting early LV dysfunction [19], as well as being an independent predictor of cardiac events [20] and postoperative survival.

The prognostic value of LV-GLS was also demonstrated in cardiomyopathies. In hypertrophic cardiomyopathy, an impaired LV-GLS was associated with myocardial fibrosis and worse outcomes [21,22], with some studies suggesting that LV-GLS may be more sensitive than cardiac magnetic resonance in detecting fibrosis [23,24]. Raafs et al. showed an incremental value of LV-GLS in patients with dilated cardiomyopathy, as an independent predictor of adverse outcomes exceeding the LVEF [25].

However, implementing GLS in the clinical decision-making algorithm poses several problems. LV-GLS, as a load-dependent parameter, in the context of the volume overload that exists in severe MR, may have different reference values compared with normative data from healthy individuals. Studies have proposed a range of values, from −17.9% to −21.7%, for prognostic preoperative cutoffs [26,27,28,29,30,31]. There is also an inter-vendor variability that makes the standardization of GLS values difficult [32,33].

Most available studies have assessed LV-GLS in a perioperative phase, but there are little data regarding its role in monitoring asymptomatic patients during a longer watchful waiting follow-up, and the cutoff values that predict the development of symptoms or the decline of the LVEF.

The main objective of this study was to assess the role of serial LV-GLS changes during follow-up in a cohort of asymptomatic patients, with different degrees of MR and to correlate these changes with a composite endpoint that consisted of the current criteria for intervention [1].

## 2. Materials and Methods

### 2.1. Study Population

This study was conducted prospectively on 218 patients with chronic asymptomatic, primary MR, grouped as mild, moderate, and severe. Patients were enrolled over an interval of four years (2019–2023) and were recruited from two centers: the Department of Internal Medicine, “Iuliu Hatieganu” University of Medicine and Pharmacy, Cluj Napoca, Romania and “Niculae Stancioiu” Heart Institute, Cluj Napoca.

Inclusion criteria were established based on a multiparametric echocardiographic assessment of MR severity according to current guidelines [1]. Table 1 lists the echocardiographic inclusion criteria that were used for patient selection.

Exclusion criteria consisted of the following: 1. symptomatic patients with a dilated left ventricular end diastolic diameter (LVEDD) > 45 mm and/or a depressed LVEF < 60% who met the criteria for intervention, 2. dilated cardiomyopathy, 3. ischemic MR, 4. hypertrophic cardiomyopathy, 5. active endocarditis, 6. connective tissue disorders, 7. more than moderate aortic valve disease, 8. reduced life expectancy, and 9. inadequate acoustic transthoracic window.

**Figure 1:**
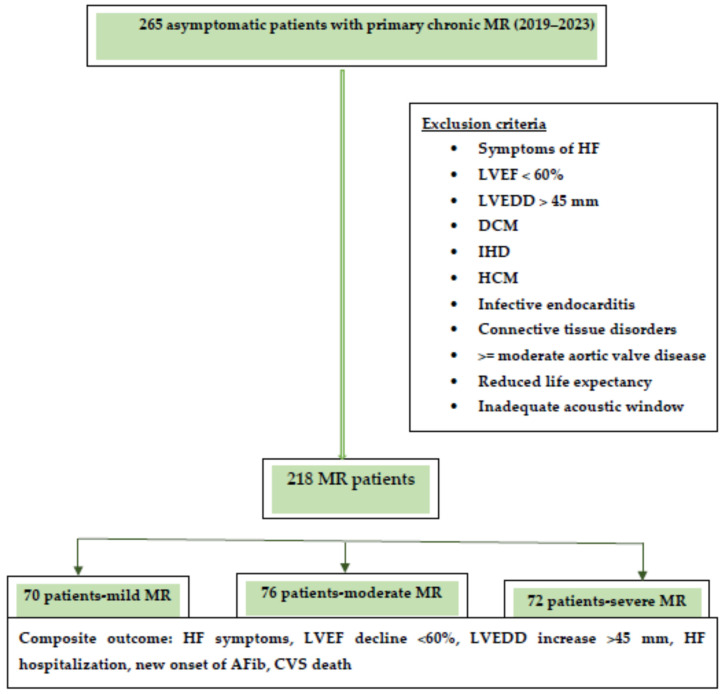
Flowchart of the study design.

The composite endpoint consisted of the occurrence of heart failure symptoms, hospitalization for heart failure, echocardiographic signs of left ventricular dysfunction (LVEF < 60% and/or LVEDD > 45 mm), newly onset atrial fibrillation, and cardiovascular death. A flowchart of the study design is presented in Figure 1. The median follow-up was 30 months.

Abbreviations: HF, heart failure; LVEF, left ventricular ejection fraction, LVEDD, left ventricular end diastolic diameter; DCM, dilated cardiomyopathy; IHD, ischemic heart disease; HCM, hypertrophic cardiomyopathy; MR, mitral regurgitation.

The symptoms were assessed according to the New York Heart Association (NYHA) classification. A twelve-lead resting ECG and blood tests—including full blood count, hematocrit, creatinine, NT-proBNP, and transthoracic echocardiogram with speckle tracking—were performed in all patients. We also recorded demographic data—including age, gender, height, weight, medical history of hypertension, diabetes, prior atrial fibrillation and chronic kidney disease, and concomitant medication.

Global longitudinal strain was measured at baseline and every 6 months during the follow-up. The relative decline in GLS was calculated as the difference between the baseline GLS and GLS at 30 months, relative to the baseline GLS.

The study was approved by the local ethics committee of the “Iuliu Hatieganu” University of Medicine and Pharmacy, Cluj Napoca (decision number 235/2018), and was conducted in accordance with the principles of the Declaration of Helsinki. All patients were informed of the study protocol and signed an informed consent form.

### 2.2. Echocardiography

Transthoracic echocardiography (TTE) was performed with a commercially available echocardiographic system (GE Vivid E90, GE Vingmed Ultrasound, Horten, Norway).

Mitral valve morphology was assessed using multiple standard transthoracic echocardiographic views, averaged Doppler measurements for three cardiac cycles in patients in sinus rhythm, and five cardiac cycles for atrial fibrillation, respectively. As per current guidelines [1], a multiparametric approach was used to assess MR severity. Vena contracta (VC) was measured as the smallest diameter distal to the regurgitant orifice, with the color Doppler Nyquist limit set to 50–70 cm/s. The proximal isovelocity surface area method (PISA), when feasible, was used to calculate the effective regurgitant orifice area (EROA), the regurgitant volume (RVol), and regurgitant fraction (RF) using a Nyquist limit of 25–40 cm/s, with the measurement of PISA radius at mid-systole. Continuous wave Doppler was used in an apical four-chamber view to measure MR peak velocity and the velocity time integral (VTI). Pulmonary veinous flow in the right upper pulmonary vein was investigated using pulsed wave Doppler.

The LVEF was calculated using the Simpson method, measuring the LV end diastolic and end systolic volumes. Pulmonary arterial pressure was derived from the tricuspid regurgitation velocity gradient and inferior vena cava size, as per ESC guidelines [1].

**Figure 2:**
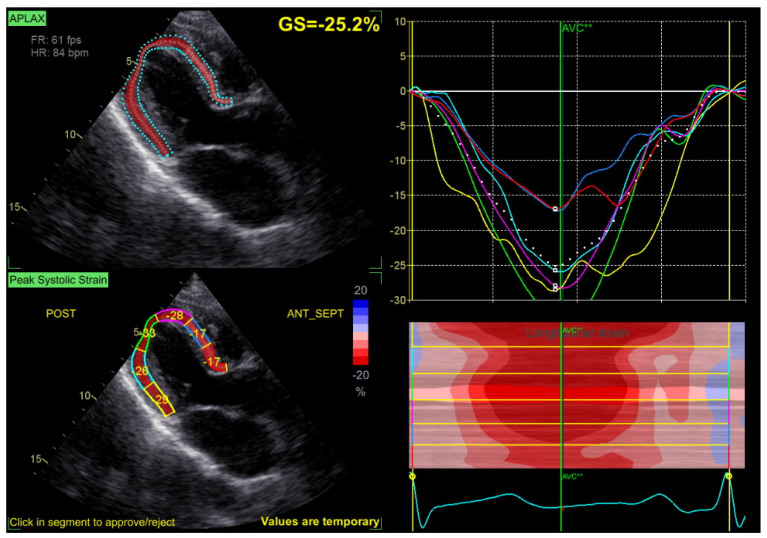
Example of a GLS measurement, with normal values of GLS at −25.2% in a patient with mild MR.

Speckle tracking analysis was performed from the apical views (long axis, 2 and 4 chambers) at a frame rate > 50 fps, with manual correction of the endocardial borders as needed. GLS was calculated by averaging the peak longitudinal strain values of the 17 segments, excluding segments that could not be traced correctly (Figure 2).

LV-GLS values are expressed, by convention, as negative numbers (normal range −19% to −20%), with smaller values reflecting better LV longitudinal function. However, in this study, we report LV-GLS using positive values that are proportional to the quality of the longitudinal LV function in order to better convey the clinical relevance of the findings.

### 2.3. Study Follow-Up

All patients underwent a follow-up appointment every six months, consisting of an assessment of clinical status and a complete echocardiographic exam, including speckle tracking imaging. Left and right ventricular dimensions and volumes, the LVEF, MR severity, estimated systolic pulmonary arterial pressure, and LV-GLS were recorded at each visit.

Patients that met the criteria for valve intervention according to the guidelines during their follow-up were referred to the cardiovascular surgeon by the treating cardiologist.

### 2.4. Statistical Analysis

Statistical analysis was performed using SPPS (version 21, Statistical Package for the Social Sciences, International Business Machines Inc., New York, NY, USA), with a *p* value < 0.05 being considered statistically significant in the analysis of variance.

Continuous patient characteristics were reported as mean +/− standard deviation when normally distributed or as medians (interquartile range) otherwise, and categorical variables were reported as frequencies and proportions. In the group comparison, the chi square or Fisher exact test were used for categorical variables, and the Kruskal–Wallis test for continuous variables. The log rank test was used to compare survival. Linear regression and logistic regression were used to determine the characteristics associated with LV-GLS and the relative decline in GLS.

Univariable survival analysis and multivariable Cox proportional hazards modeling were performed to determine the characteristics associated with the composite outcome.

## 3. Results

### 3.1. Baseline Characteristics

A cohort of 218 asymptomatic patients with chronic primary MR were enrolled from 2019 to 2023. According to MR severity, patients were divided into three subgroups: 70 patients with mild MR, 76 patients with moderate MR, and 72 patients with severe MR.

Baseline characteristics of all patients are summarized in Table 2.

Patients with severe and moderate MR had a significantly higher rate of atrial fibrillation, higher levels of NT-proBNP, and were more frequently treated with SGLT2 inhibitors. The echocardiographic parameters of the study groups are listed in Table 3.

### 3.2. GLS at Baseline and during Follow-Up

At baseline, mean GLS values were significantly lower in patients with moderate (19.5%) and severe MR (19.1%) versus those with mild MR (22.3%), despite a normal value of the LVEF in all groups (*p* < 0.01).

**Figure 3:**
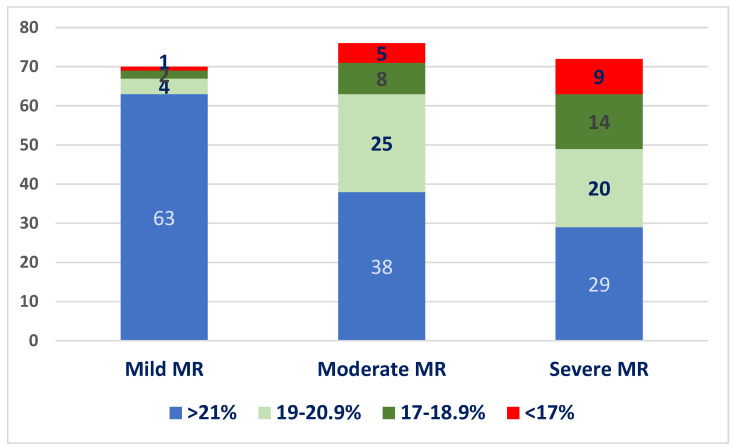
Distribution of baseline LV-GLS values in the three subgroups.

We divided patients into four quartiles according to GLS values: GLS > 21%, 20.0–19%, 18.9–16%, and <16%. The distribution of patients into these quartiles at baseline, in each MR severity subgroup, is illustrated in Figure 3. Most patients with mild MR had normal GLS values at baseline (90%), whereas only 52% of patients with moderate MR and 40.2% of the patients with severe MR had normal baseline GLS values.

Mean LV-GLS decreased in all three subgroups during the follow-up. In patients with mild MR, this decrease was apparent later during the follow-up (at 24 months) compared with patients with moderate and severe MR, with both of these groups showing a significant decline in mean GLS during the first 12 months of follow-up (*p* < 0.01).

**Figure 4:**
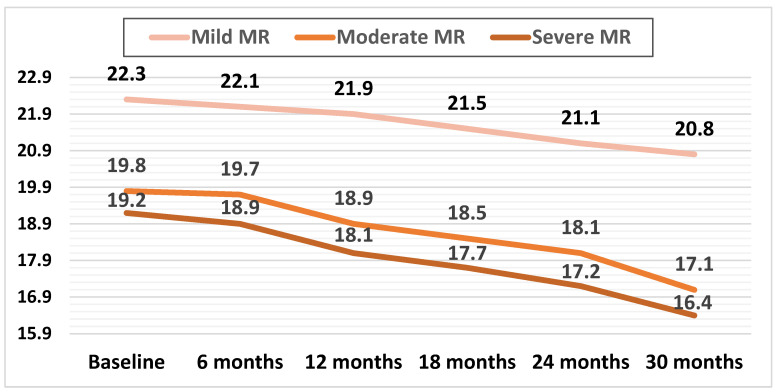
Mean values of LV-GLS during the follow-up in the three subgroups.

The mean GLS values during the follow-up are illustrated in Figure 4.

The relative decrease in GLS was calculated as the decrease in GLS at 30 months divided by the baseline LV-GLS. The relative decrease in mean LV-GLS was significantly higher in the subgroups with moderate and severe MR, at 13.6% (*p* < 0.01) and 14.5% (*p* < 0.01), respectively, compared to the mild MR subgroup, at 6.72%.

**Figure 5:**
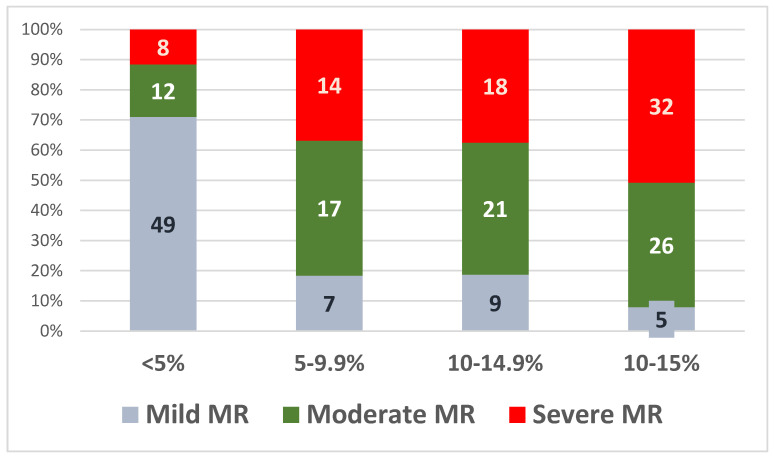
Relative LV-GLS decline at 30 months in the three subgroups.

The values of the relative LV-GLS decline at 30 months were then divided into four quartiles: 5%, 5–9.9%, 10–14.9%, and >15%, as shown in Figure 5.

### 3.3. Clinical Outcomes and Follow-Up

During a median follow-up of 30 months, a total of 31 (8.7%) patients met the composite outcome.

A decrease in the LVEF of < 60% was noted in nineteen patients, fifteen of which developed symptoms and four remain asymptomatic. Only four patients required hospitalization for heart failure symptoms. An increase in the LVEDD of >45% was noted in 11 patients. New onset atrial fibrillation occurred in eight patients. There were no cardiovascular deaths.

The incidence of the composite endpoint was significantly higher in patients with severe (17 patients, 23.6%) and moderate MR (11 patients, 14.4%) compared to mild MR (three patients, 4.2%) (*p* = 0.021).

Using univariate analysis and multivariate Cox regression analysis, we found two independent predictors of the composite outcome: a baseline GLS < 18% (HR = 1.59, 95% CI 1.17 to 2.86, *p* < 0.01) and a relative decrease in GLS > 10% (HR = 1.74, 95% CI 1.2 to 2.91, *p* < 0.01).

**Figure 6:**
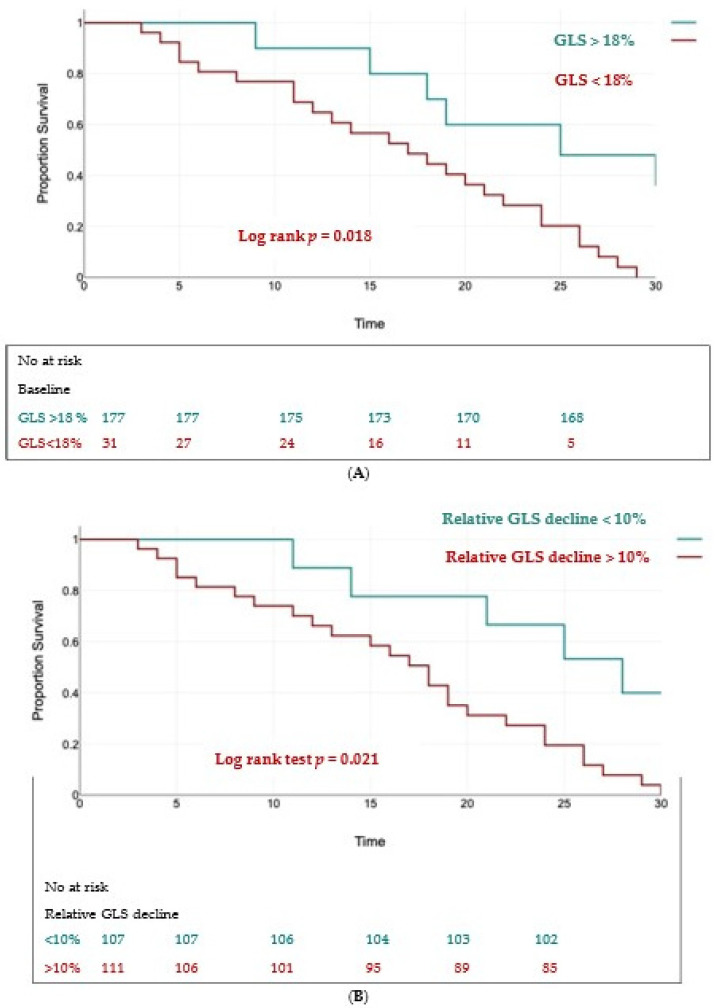
Kaplan–Meier survival curves for event-free survival in patients with baseline LV-GLS < 18% (panel (**A**)) and a relative decline in LV-GLS > 10% (panel (**B**)).

The Kaplan–Meier curves for event-free survival showed significantly higher rates of MACEs in patients with a baseline GLS < 18% (log rank test, *p* = 0.018) (Figure 6A) and in those with a relative decrease in GLS > 10% (log rank test, *p* = 0.021) (Figure 6B).

## 4. Discussion

In order to prevent the decline of the LVEF and a poor postoperative outcome, surgery in severe MR should be timed before the development of irreversible LV dysfunction [1,34]. In severe MR, the LVEF poorly reflects the systolic function of the LV, remaining “normal” for a long time, despite the subclinical decline in LV systolic function. Therefore, there is a need for more sensitive markers of LV dysfunction to help optimize the timing of intervention.

LV-GLS measured by speckle tracking imaging is a sensitive and specific marker for LV systolic longitudinal dysfunction [35], being less load-dependent than the LVEF, and it has been shown to be a more sensitive predictor for LV systolic dysfunction than the decline in LVEF [7,36].

Most studies have assessed the prognostic value of LV-GLS in patients who underwent MV intervention. Even in asymptomatic patients with a preserved LVEF, a LV-GLS < 20.7% was independently associated with higher mortality [37]. Alashi et al. also described the incremental prognostic utility of NT-proBNP to LV-GLS [37].

Hiemstra et al. showed that patients with the most preserved LV-GLS (>22.3%) had the best outcome in terms of all-cause mortality, whereas those with LV-GLS < 19.5% had the worst survival at a 5 and 10 year postoperative follow-up [27], with an incremental value of LV-GLS over clinical risk factors for long-term survival.

Moreover, LV-GLS has emerged as an independent predictor of LV remodeling and LV dysfunction in long-term follow-ups [38,39]. A baseline LV-GLS < 18% [29] and 17.9%, respectively [40], was predictive of an early 10% decline in the postoperative LVEF. LV-GLS < 19.9% was the strongest independent predictor for long-term LV dysfunction [39].

Lancelotti et al. showed the incremental prognostic value of exercise LV-GLS in asymptomatic MR patients [41], suggesting that during exercise, there is a lack of longitudinal contractile reserve despite the lack of symptoms. The reduction in LV-GLS during exercise and LV-GLS at peak exercise were predictive of LV dysfunction.

However, the predictive postoperative LV-GLS cutoffs varied significantly between studies, ranging between −17.9% and −21.7% [29,37,38,42]. Additionally, normal GLS values have also varied largely across studies due to patient-related (age, gender, ethnicity) and hemodynamic factors (heart rate and blood pressure) [43,44,45]. These aspects have made it difficult to implement LV-GLS in the follow-up of patients with severe MR before surgery.

Therefore, a relative decline in baseline LV-GLS values could be more useful than the absolute LV-GLS values during the follow-up of asymptomatic patients with severe MR.

In the present study, we assessed LV-GLS at baseline and every six months during a median follow-up period of 30 months in a large cohort with various degrees of MR, and correlated the relative decline in LV-GLS with a composite outcome that required valve intervention.

Despite the lack of significant differences in the LVEF at baseline between groups, asymptomatic patients with moderate and severe MR displayed a lower mean LV-GLS than those with mild MR (19.5% and 19.1% versus 22.3%, respectively, *p*, 0.05). Almost half of the patients with moderate (48%) and severe MR (52%) had abnormal baseline LV-GLS (<21%).

This is consistent with data reported by other studies [29,37] and underlines the presence of longitudinal dysfunction in patients that do not meet the current guideline criteria for intervention The substrate for this longitudinal dysfunction is probably myocardial fibrosis. A study conducted by Liu et al. in symptomatic and asymptomatic MR patients undergoing intervention combined cardiac magnetic resonance and myocardial biopsies in investigating myocardial fibrosis. They documented the presence of myocardial fibrosis even in asymptomatic MR patients, with no significant difference in fibrosis according to the NYHA classification [46]. Different patterns of fibrosis were described: interstitial, perivascular, and replacement fibrosis.

LV-GLS was correlated more closely with myocardial interstitial fibrosis than the LVEF [47], which was also seen in animal models.

During the follow-up, patients with moderate and severe MR showed a constant decline in LV-GLS, more evident after 12 months (Figure 4). Of note, patients with mild MR also showed a decline in LV-GLS, but this was only apparent after 24 months. The relative decline in mean LV-GLS was also significantly higher in patients with moderate and severe MR versus mild MR (13.6% and 14.5%, respectively, versus 6.72%).

The steady decline in LV-GLS could be explained by the development of progressive, interstitial, and replacement fibrosis secondary to the volume overload as suggested by cardiac magnetic resonance tissue characterization studies [48]. In animal models, severe volume overload in MR causes elongation of the cardiomyocytes along the longitudinal axis, disorganization, and finally, disruption of the architectural myocardial structure, with the activation of cardiac fibroblasts, the progressive expansion of the extracellular matrix, and interstitial remodeling [49].

Following the model of the LV-GLS follow-up for the cardiac toxicity of chemotherapy in oncologic patients [50], we divided the relative decline of GLS in four quartiles (from <5% decline in relative LV-GLS to >15% decline). Most patients in which a relative decline of >10% in LV-GLS was noted, had moderate or severe MR (97 patients, 87%). These results suggest that not only subclinical longitudinal LV dysfunction is already present in a large number of asymptomatic MR patients with a preserved LVEF, but that this dysfunction is progressive during the follow-up and more severe in moderate and severe MR.

In our study, a cutoff value of 18% for LV-GLS was predictive of the composite outcome, which translated into the need for valve intervention. But, more importantly, a relative decrease in LV-GLS more that 10% had a higher predictive value for the composite outcome.

The etiology of primary chronic MR in our cohort was heterogenous, including both patients with Barlow’s disease and fibroelastic deficiency. We did not differentiate between these etiologies in the statistical analysis of LV-GLS data. One of the few studies including etiology in the multivariate analysis did not find an association with long-term mortality or cardiovascular events [27].

Considering that patients with Barlow’s disease are younger, with tissue excess and thickening, usually with multiple prolapsing segments frequently with chordal elongation, whereas patients with fibroelastic deficiency are older (>60 years), with a normal quantity of leaflet tissue, a normal-sized annulus, elongation and/or rupture of one or more chordae in a single prolapsing segment; the long-term prognosis and LV dysfunction progression may differ between these subgroups. Future studies are needed to refine the follow-up strategy and intervention according to etiology.

## 5. Limitations

This was a prospective, observational study, with a heterogenous cohort of patients with different mechanisms of MR. There may be a difference in the significance of LV-GLS values in different etiologies of MR. Prospective, larger, controlled trials are needed to establish cutoff values for LV-GLS that can be introduced in clinical practice.

## 6. Conclusions

LV–GLS is a reliable marker of subclinical LV dysfunction, which could be used in the follow-up of asymptomatic MR patients to optimize the timing of intervention before irreversible LV dysfunction occurs. The results of this study suggest that a baseline LV-GLS less than 18% or a relative decline in LV-GLS of more than 10% from the baseline could be predictive for the need for valve intervention; however, larger randomized studies are needed in order to establish reliable cutoff values for clinical practice.

## Figures and Tables

**Table 1:** Multiparametric echocardiographic parameters used for MR severity assessment of patients, including criteria modified from [1].

	Mild MR	Moderate MR	Severe MR
Qualitative parameters			
Mitral valve morphology	normal/modified	normal/modified	mitral valve severe prolapse/flail papillary muscle rupture or perforation
MR jet-Color Doppler	small, central	Intermediate	large, central/ eccentric
Proximal convergence area	Absent/small	Intermediate	Large
Continuous wave Doppler	low signal, parabolic shape	parabolic shape	high intensity, triangular shape
Semiquantitative parameters			
Vena contracta (mm)	<3	3–6	≥7
Pulmonary venous flow	Dominant S wave	Low velocity S wave(S < D)	systolic flow reversal
Mitral Doppler inflow	dominant A wave	Variable	Dominant E wave (>1.5 m/s)
Mitral VTI/Aortic VTI	<1	Intermediate	>1.4
Quantitative parameters			
EORA (mm^2^)	<20	20–39	≥40
RVol (ml)	<30	30–59	≥60

Abbreviations: MR, mitral regurgitation; VTI, velocity time integral; S, systolic; D, diastolic; EORA, effective orifice area; RVol, regurgitant volume.

**Table 2:** Baseline characteristics of participants.

Clinical Data	Mild MR (n = 70)	Moderate MR (n = 76)	Severe MR (n = 72)	*p* Value (Mild vs. Moderate MR/Mild vs. Severe MR)
Age (years), mean, SD	62.1 ± 10.9	63.9 ± 11.7	64.2 ± 9.4	0.615/0.714
Male sex, n (%)	36 (51.4)	41(53.9)	38 (52.7)	0.391/0.281
Body mass index (kg/m^2^), SD	24.3, 4.6	25.2, 4.9	25.6, 4.7	0.416/0.522
NYHA Class I/II/III	58/12/0	45/31/0	38/34/0	0.218/0.194
**Comorbidities**				
Hypertension, n (%)	34 (48.5)	39 (51.3)	38 (52.7)	0.291/0.398
Diabetes	22 (30.5)	25 (32.8)	24 (33.3)	0.335/0.571
Atrial fibrillation	5 (7.1)	9 (11.8)	12 (13.8)	0.033/0.039
Chronic kidney disease	8 (11.4)	10 (13.1)	11 (15.2)	0.173/0.357
**Blood tests**				
Hemoglobin (g/dL)	12.8 ± 1.7	12.7 ± 1.4	12.7 ± 1.6	0.877/0.819
NT-proBNP (pg/mL), SD	75 ± 11.8	148 ± 12.7	387 ± 15.9	0.006/0.005
eGFR (mL/min/1.73 m2), SD	80 ± 11.7	77 ± 13.1	78 ± 12.2	0.199/0.185
**Medication**				
ACEIs/ARBs/ARNI, n (%)	33 (47.1)	37 (48.6)	34 (37.2)	0.186/0.374
β-blockers, n (%)	38 (54.2)	40 (52.6)	41 (56.9)	0.381/0.412
SGLT2 inhibitors	17 (14.2)	24 (31.5)	27 (37.5)	0.028/0.021
MRAs	9 (12.8)	12 (15.7)	16 (22.2)	0.217/0.181
Diuretics	10 (14.2)	11 (14.4)	13 (18.0)	0.411/0.429

Abbreviations: SD, standard deviation; NYHA, New York Heart Association; NT-proBNP, N-terminal brain natriuretic peptide; eGFR, estimated glomerular filtration rate; ACEI, angiotensin conversion enzyme inhibitor; ARB, angiotensin receptor blocker; ARNI, angiotensin receptor–neprilysin inhibitor; MRA, mineralocorticoid receptor antagonist; SGLT2, sodium–glucose cotransporter.

**Table 3:** Echocardiographic parameters.

Parameters Transthoracic Echocardiography	Mild MR (n = 70)	Moderate MR (n = 76)	Severe MR (n = 72)	*p* Value (Mild vs. Moderate MR/Mild vs. Severe MR)
LVEDD, mm	45 ± 11.2	47 ± 12.2	51 ± 8.1	0.187/0.119
LVESD, mm	26 ± 7.7	29 ± 10.8	33 ± 12.6	0.201/0.168
LVEDV, mL	137 ± 23.1	158 ± 18.3 1	195 ± 22.7	0.097/0.81
LVESV, mL	65 ± 14.3	78 ± 11.7	86 ± 12.8	0.092/0.086
LVEDDi, mm/m^2^	26 ± 6.3	35 ± 10.4	37 ± 11.2	0.168/0.116
LVESDi, mm/m^2^	14 ± 8.1	16 ± 9.6	17 ± 8.5	0.374/0.276
LVEDVi mL/m^2^	68 ± 13.1	71.6 ± 12.7	85.3 ± 14.1	0.031/0.028
LVESVi mL/m^2^	35 ± 7.8	39 ± 11.2	42 ± 10.3	0.199/0.082
LVEF %	68 ± 7	67 ± 5	67 ± 8	0.817/0.837
LVS GLS, %	22. 3 ± 2.1	19.5 ± 3.1	19.1 ± 3.7	0.024/0.019
LAVoli, mL/m^2^	29 ± 12.1	38 ± 14.4	45 ± 13.8	0.019/0.014
EROA, cm^2^	0.12 ± 0.05	0.24 ± 0.04	0.38 ± 0.07	0.015/0.012
RVol, mL	12.1 ± 4.7	33 ± 9.5	47 ± 11.1	0.027/0.016
RF, %	11.9 ± 4.2	32 ± 7.9	48 ± 9.3	0.001/0.001
PSAP, mmHg	26 ± 5.2	32 ± 8.1	37 ± 10.4	0.008/0.007
TAPSE, mm	31 ± 3.8	29 ± 4.1	29 ± 3.8	0.277/0.2.74

Abbreviations: LVEDD, left ventricular end diameter; LVESD, left ventricular end systolic diameter; LVEDV, left ventricular end-diastolic volume; LVESV, left ventricular end systolic volume; LVEDDi, left ventricular end diameter index; LVESDi, left ventricular end systolic diameter index; LVEDVi, left ventricular end diastolic volume index; LVESVi, left ventricular end systolic volume index; LVEF, left ventricular ejection fraction; LV-GLS, left ventricular global longitudinal strain; LAVoli, left atrial volume index; EROA, effective regurgitant orifice area; RVol, regurgitant volume; RF, regurgitant fraction; TAPSE, tricuspid annular systolic excursion.

## Data Availability

The data presented in this study are available upon request from the corresponding author.
